# HIV Infection is associated with compositional and functional shifts in the rectal mucosal microbiota

**DOI:** 10.1186/2049-2618-1-26

**Published:** 2013-10-12

**Authors:** Ian H McHardy, Xiaoxiao Li, Maomeng Tong, Paul Ruegger, Jonathan Jacobs, James Borneman, Peter Anton, Jonathan Braun

**Affiliations:** 1 Pathology and Laboratory Medicine, UCLA, 10833 Le Conte Ave 13-188 CHS, Los Angeles, CA 90095, USA; 2 Inflammatory Bowel Disease & Immunobiology Research Institute, Cedars Sinai Medical Center, Los Angeles 90048, USA; 3 Pharmacology, UCLA, Los Angeles, CA, USA; 4 Plant Pathology, UC Riverside, Riverside, CA, USA; 5 Department of Medicine, UCLA, Los Angeles, CA, USA; 6 Center for HIV Prevention Research, UCLA AIDS Institute, Los Angeles, CA, USA

**Keywords:** HIV, Microbiome, Metagenome, Mucosa

## Abstract

**Background:**

Regardless of infection route, the intestine is the primary site for HIV-1 infection establishment and results in significant mucosal CD4+ T lymphocyte depletion, induces an inflammatory state that propagates viral dissemination, facilitates microbial translocation, and fosters establishment of one of the largest HIV reservoirs. Here we test the prediction that HIV infection modifies the composition and function of the mucosal commensal microbiota.

**Results:**

Rectal mucosal microbiota were collected from human subjects using a sponge-based sampling methodology. Samples were collected from 20 HIV-positive men not receiving combination anti-retroviral therapy (cART), 20 HIV-positive men on cART and 20 healthy, HIV-negative men. Microbial composition of samples was analyzed using barcoded 16S Illumina deep sequencing (85,900 reads per sample after processing). Microbial metagenomic information for the samples was imputed using the bioinformatic tools PICRUST and HUMAnN. Microbial composition and imputed function in HIV-positive individuals not receiving cART was significantly different from HIV-negative individuals. Genera including *Roseburia*, *Coprococcus*, *Ruminococcus*, *Eubacterium*, *Alistipes* and *Lachnospira* were depleted in HIV-infected subjects not receiving cART, while *Fusobacteria*, *Anaerococcus*, *Peptostreptococcus* and *Porphyromonas* were significantly enriched. HIV-positive subjects receiving cART exhibited similar depletion and enrichment for these genera, but were of intermediate magnitude and did not achieve statistical significance. Imputed metagenomic functions, including amino acid metabolism, vitamin biosynthesis, and siderophore biosynthesis differed significantly between healthy controls and HIV-infected subjects not receiving cART.

**Conclusions:**

HIV infection was associated with rectal mucosal changes in microbiota composition and imputed function that cART failed to completely reverse. HIV infection was associated with depletion of some commensal species and enrichment of a few opportunistic pathogens. Many imputed metagenomic functions differed between samples from HIV-negative and HIV-positive subjects not receiving cART, possibly reflecting mucosal metabolic changes associated with HIV infection. Such functional pathways may represent novel interventional targets for HIV therapy if normalizing the microbial composition or functional activity of the microbiota proves therapeutically useful.

## Background

HIV-1 transmission and replication occur primarily at mucosal sites. There is increasing recognition that HIV-1 infection is substantially a mucosal disease with systemic manifestations [1]. Regardless of infection route, gut-associated lymphoid tissue (GALT) is the major site of virus replication early in HIV infection due to local retention, enhanced activation states and increased memory immune function of GALT CD4+ T lymphocytes as compared to peripheral blood mononuclear cells (PBMCs) [2-4]. Accordingly, acute HIV infection results in massive depletion of GALT CD4+ T lymphocytes with slow and only partial reconstitution with combination anti-retroviral therapy (cART) [1,5-8]. Other reports have indicated complete intestinal reconstitution of CD4+ T cells in subjects receiving cART that have sustained undetectable HIV replication for many years [9]. In health, lymphocyte-mediated inflammatory processes naturally occur, in part, as a response to ongoing luminal antigenic stimulation [10] and help shape immune and inflammatory responses [11,12]. However, HIV-induced immunological imbalance results in pathological manifestations including first acute and then chronic mucosal tissue inflammation, systemic immunological activation, increased epithelial permeability and systemic microbial translocation [13-16]. Indeed, local mucosal (and distant, peripheral) inflammation has emerged as a key process in HIV infection, dissemination, pathogenesis and possibly perpetuation [17]. Thus, strategies to reverse or reduce HIV-specific as well as more generalized subsequent inflammation could help prevent HIV infection sequelae and dissemination.

One potential source of such inflammation is intestinal bacteria. Commensal microbiota have major effects on the biologic state of the host cell types in the mucosal compartment. They modulate epithelial processes controlling stem cell replenishment, barrier permeability and microbial intrusion [18,19], mucosal lymphocyte development and IL-17- and IL-22- dependent immune surveillance to microbial challenge [18,20,21], and mucosal myeloid (macrophage, dendritic cell, and neutrophil) microbial surveillance and immune regulation [22-25]. Given that HIV is associated with intestinal inflammation and that intestinal microbiota can be altered in inflammatory diseases, intestinal microbial compositional aberrations might be expected in HIV-infected individuals [26,27]. However, only minor differences in abundance of a few specific pathogens have been observed in HIV-infected human feces [28], and larger-scale studies of fecal microbiota involving simian immunodeficiency virus (SIV) indicate no significant compositional differences from healthy controls [29,30]. The discrepancy between expectations and published observations could indicate that: 1) no significant changes in microbial composition occur in HIV-infected subjects, and 2) any major changes in microbial composition of HIV-infected subjects are minor and/or easily masked. The latter might be possible if, for example, mucosal, as opposed to luminal, bacteria were differentially abundant in HIV-infected subjects.

The relative inaccessibility of the intestine for direct sampling has historically made fecal sampling from stool the standard for investigating such associations. While informative, some studies indicate that human fecal microbiota composition from stool differs from that of the intestinal mucosa [26,31,32], suggesting potentially different grooming mechanisms. Accordingly, methods of collecting unperturbed mucosal bacteria are highly desirable, as mucosal communities might interact more intimately with the host mucosal immune system [33].

Therefore, to investigate our hypotheses that HIV infection is associated with altered intestinal microbial composition, we utilized a rectal mucosal sampling strategy involving small, anally-inserted sponges to absorb mucosal-derived bacteria. These samples, collected from three human cohorts, enabled assessment of whether the intestinal mucosal microbiome composition or function varied with HIV infection and allowed investigation of the ecologic influence of cART.

## Methods

### Subject recruitment

The protocol was designed by the investigators and approved by the UCLA Office of the Human Research Protection Program Institutional Review Board (UCLA IRBs #11-001592 and #10-000750) with all participants providing written informed consent. Protocol-based inclusion criteria required that only men age ≥18 years were recruited into this pilot study. Subjects were divided into three groups: 20 healthy HIV-negative control subjects; 20 healthy HIV-positive subjects on chronic cART; and 20 healthy HIV-positive subjects not on cART (cART-naïve or not on cART for ≥3 months). Exclusion criteria included: being female; having history of inflammatory bowel disease (IBD); having any active inflammatory conditions affecting the rectum; and use of rectally administered medications, including over-the-counter enemas, within 48 hours.

### Sample procurement

All subjects (as specified above) were seen once in the UCLA Digestive Diseases Clinic for sample collection. Following a brief history, physical examination and confirmation of inclusion/exclusion criteria, as well as confirmation of subject-reported HIV serostatus (PCR and HIV-1 antibody test), subjects received a clinician-applied preparatory enema (118-ml saline enema). Subjects were asked to retain the fluid for at least 5 minutes and then expel the fluid into a toilet. While this procedure could conceivably disturb mucosal surface contents, it was deemed necessary to eliminate stool that might otherwise interfere with sponge placement. Following a 15 minute rest, mucosal sampling by sponge collection took place, using previously reported methods used for secreted antibodies and cytokines [34,35]. Briefly, two ophthalmic eye spears (Beaver Visitec, Waltham, MA, USA) were simultaneously inserted into the rectum via anoscope, as previously reported [34,35], and allowed to absorb mucosal material for 5 minutes. Samples were immediately placed on ice and transported to the laboratory for immediate processing.

### Sample preparation and 16S V4 sequencing analysis

Sponges were removed from their plastic stems and individually placed in 0.5-ml tubes (Eppendorf, Hauppauge, NY, USA), which had the distal end previously pierced using a sterile 18-gauge needle (BD Biosciences, San Jose, CA, USA); each of these individual tubes were then placed into 2-ml tubes (Eppendorf, Hauppauge, NY, USA). Bacteria were quickly eluted and pelleted by adding 100 μl of 25 mM HEPES, 50 mM NaCl, 1% Triton-X, 1 mM DTT, and 5 mM EDTA and centrifuging in an Eppendorf 5415D centrifuge (Eppendorf, Hauppauge, NY, USA) for 30 s. This collection step was repeated with another 100 μl of the elution buffer above. Supernatant was immediately removed and pellets were frozen in a -80° freezer (Model: ELT1786-9-D40, Thermo Scientific, Asheville, NC, USA) with a backup phone system, until further processing.

Genomic DNA was extracted from the 60 samples using the PowerSoil DNA Isolation Kit (MO BIO Laboratories, Carlsbad, CA, USA), and a 30-second beat-beating step using a Mini-Beadbeater-16 (BioSpec Products, Bartlesville, OK, USA). High-throughput sequencing analysis of bacterial rRNA genes was performed using extracted genomic DNA as the templates. One hundred microliter amplification reactions were performed in an MJ Research PTC-200 thermal cycler (Bio-Rad Inc., Hercules, CA, USA) and contained: 50 mM Tris (pH 8.3), 500 μg/ml bovine serum albumin (BSA), 2.5 mM MgCl2, 250 μM of each deoxynucleotide triphosphate (dNTP), 400 nM of each primer, 4 μl of DNA template, and 2.5 units JumpStart Taq DNA polymerase (Sigma-Aldrich, St Louis, MO, USA). The PCR primers (F515/R806) targeted a portion of the 16S rRNA gene containing the hypervariable V4 region, with the reverse primers including a 12-bp barcode [36]. All primer sequences are available in Additional file 1. Thermal cycling parameters were 94°C for 5 minutes; 35 cycles of 94°C for 20 seconds, 50°C for 20 seconds, and 72°C for 30 seconds, followed by 72°C for 5 minutes. PCR products were purified using a MinElute 96 UF PCR Purification Kit (Qiagen, Valencia, CA, USA). DNA sequencing was performed using an Illumina HiSeq 2000 (Illumina, Inc., San Diego, CA, USA). Clusters were created using template concentrations of 1.9 pM and PhiX at 65 K/mm^2^, (manufacturer’s recommendations for samples with uneven distributions of A, C, G and T). One hundred base sequencing reads of the 5′ end of the amplicons and seven base barcode reads were obtained using the sequencing primers listed in Additional file 1. De-multiplexing, quality control, and operational taxonomic unit (OTU) binning were performed using Quantitative Insights into Microbial Ecology (QIIME) [37].

The total initial number of sequencing reads was 71,581,480. Low quality sequences were removed using the following parameters: Q20, minimum number of consecutive high-quality base calls = 100 bp, maximum number of N characters allowed = 1, maximum number of consecutive low-quality base calls allowed before truncating a read = 3. Numbers of sequences removed using the aforementioned quality control parameters were: barcode not in mapping file (35,296,547), reads too short after quality truncation (4,926,462), and too many Ns (5,431). Remaining reads numbered 31,353,040, which were then used to pick OTUs from the GreenGenes reference database (May 18, 2012 database); this database automatically bins OTUs at 97% identity, ensuring the resulting data were compatible with phylotypic investigation of communities by reconstruction of unobserved states (PICRUSt) analysis. Due to alignment failure, an additional 1,511,116 reads were discarded during OTU picking, providing 29,841,924 reads for downstream analysis.

### Bioinformatic analysis

#### Rarefaction and diversity analysis

After picking OTUs from the GreenGenes reference database, rarefaction was performed to 85,900 (corresponding to the sample with the fewest reads) reads per sample using the QIIME software suite (version 1.6) running on an Ubuntu virtual machine [37]. Alpha diversity metrics used included Phylogenetic Diversity, Chao1, observed species and Shannon index. For all sampling depths, each plotted point represents the average of ten random samplings. The comparison of alpha diversity between the three groups was performed using the two-sided Student *t*-test. Beta diversity analysis was performed in QIIME and utilized unweighted UniFrac distances to estimate sample distributions. Adonis significance analysis was performed for each pairwise comparison of sample groups using the Adonis function from the vegan R package [38].

#### Taxonomic analyses

Microbial composition at each taxonomic level was defined using the summarize_taxa function in QIIME. Prior to all analysis of variance (ANOVA), taxa at each taxonomic level were thresholded such that any taxon present in fewer than 20% of samples was discarded.

#### Statistical analysis

All statistical analyses were conducted using R (http://www.r-project.org/). HIV- and cART-associated microbial changes were calculated using ANOVA with multiple comparison correction using *q*-values (R package qvalue). Associations between imputed metagenomic functions and HIV infection were calculated using Kruskal-Wallis ANOVA and corrected for multiple comparisons using *q*-values. All taxonomic associations reported were significant *q* <0.15 unless stated otherwise. All metagenomic associations reported were significant at *q* <0.25.

#### Comparison of microbial composition with respect to cART drug classes

For this analysis, subjects were assigned to one or more groups based on the classes of prescribed cART drugs. Genus-level taxa were then analyzed for differences between subjects from each individual group compared with all cART(-) subjects. For each ANOVA comparison, genera that were not present in at least 20% of cART(+) subjects were first removed.

#### Metagenomic imputation

PICRUSt is a well-documented tool designed to impute metagenomic information based on 16S input data (http://picrust.github.io/picrust). Sample metagenomic imputation was performed using the default settings of PICRUSt (version 0.9.1). The resulting metagenomic data were then entered into the HMP unified metabolic analysis network (HUMAnN) pipeline (version 0.98) [39] to sort individual genes into Kyoto encyclopedia of genes and genomes (KEGG) pathways representing varying proportions of each imputed sample metagenome. Both PICRUSt and HUMAnN analyses were performed using the terminal interface of a QIIME virtual machine running the Ubuntu operating system.

## Results

### Rectal mucosa microbial sampling strategy

**Figure 1:**
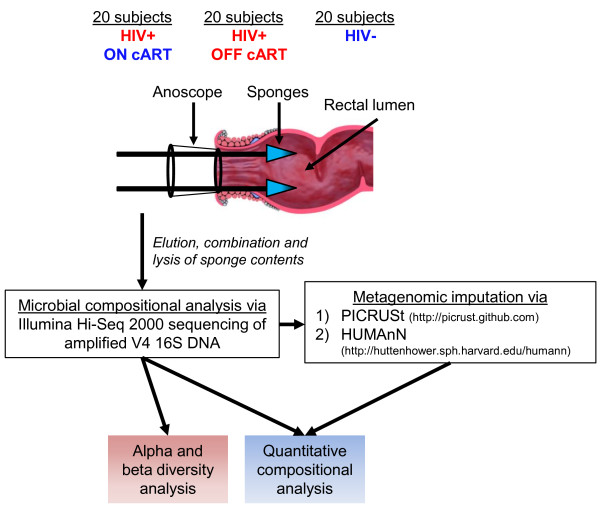
**Schematic of sampling and bioinformatic methodology.** Rectal mucosa secretions were collected from 60 human subjects as shown and subjected to high-throughput deep V4 16S sequencing. Alpha and beta diversity analyses were then performed, which suggested microbial composition was altered in HIV-infected subjects who were not receiving combination anti-retroviral therapy (cART). Microbial differences in these subjects were identified and compared with HIV-infected subjects receiving cART. In addition, the different classes of cART were analyzed to determine whether any class was significantly associated with differences in microbial composition. Imputed metagenomic differences between HIV-infected subjects not receiving cART and healthy control subjects were then identified and compared between the three patient cohorts. PICRUSt, phylotypic investigation of communities by reconstruction of unobserved states; HUMAnN, HMP unified metabolic analysis network.

In place of bowel preparation, all subjects first received a saline enema to void solid fecal contents from the rectal vault. Two absorbent ophthalmic sponges were then applied, under direct vision via anoscope, to opposing sides of the rectal mucosa (Figure 1), enabling collection of measureable levels of bacteria, protein and metabolites. Despite their small volume, eluate from each sponge allowed recovery of approximately 5 × 10^7^ ± 4.4 × SD 10^7^ of bacterial cells and approximately 10^5^ μg ± SD 75.9 of protein via bacterial hemocytometer and Bradford assay, respectively. To account for possible micro-biogeographic variations in microbial composition or abundance, material obtained from two sponges from each subject were pooled for further analyses. We did not attempt to determine whether this sampling methodology artificially enriched or depleted certain microbes, proteins, or metabolites, so we could not eliminate the possibility.

Samples were collected from 20 healthy controls (HC), 20 healthy HIV-positives on cART (cART(+)) and 20 healthy HIV-positive subjects off cART (cART(-)) for at least three months (Table 1). All collected subject metadata including age, ethnicity, viral loads, serum CD4+ T cell counts, durations of infection, and prescribed cART drug classes are tabulated in Table 1. Importantly, to eliminate the potential influences of gender, only men were recruited for this pilot study. The specific cART drugs prescribed to cART(+) subjects are tabulated in Additional file 2. Microbial components were lysed and analyzed for V4 16S composition using barcoded Illumina-HiSeq 2000 V4 sequencing. OTUs were then picked at a similarity threshold of 97% using the GreenGenes reference database, such that 497,365 ± SD 119,950 reads were retained for each sample. Sequenced reads were then rarified to 85,900 reads per sample, yielding a total of 3,281 OTUs.

**Table 1:** Human subject metadata

	**cART(-)**	**cART(+)**	**HC**	
n	20	20	20	**Subject metadata**
Gender	All male	All male	All male	
Age, years	40.9 (± 11.4)	46.3 (± 9.2)	48.6 (±12)	
Viral Loads	158,147 (± 366,197)	3,563 (± 14,333)	N/A	
Years infected	8.9 (± 8.8)	14.2 (± 6.5)	N/A	
Serum CD4 levels	439.6 (± 271.8)	534 (± 246)	NT	
Hispanic	4	1	5	**Ethnicity**
Black	13	14	9	
Caucasian	3	5	5	
Other	0	0	1	
NNRTI	N/A	9	N/A	**cART drugs prescribed**
NRTI	N/A	18	N/A	
PI	N/A	12	N/A	
II (raltegravir)	-	4	-	

Results are presented as number or mean (± SD). (cART(-), healthy HIV-positive men off combination anti-retroviral therapy (cART) for at least three months; cART(+), healthy HIV-positive men on (cART); HC, healthy controls; NT, not tested; N/A, not applicable; NNRTI, non-nucleoside reverse transcriptase inhibitor; NRTI, nucleoside reverse transcriptase inhibitor; PI; protease inhibitor; II, integrase inhibitor.

### HIV infection alters mucosal microbial diversity

To determine whether HIV infection altered microbial diversity of the rectal mucosa, alpha and beta diversity metrics were analyzed. Alpha diversity is a measure of sample-level species richness, with healthy subjects typically exhibiting more species richness (higher alpha diversity) than those with intestinal conditions, such as IBD, or obesity [40,41]. Beta diversity describes inter-subject similarity of microbial composition and facilitates identification of broad differences between samples [42].

**Figure 2:**
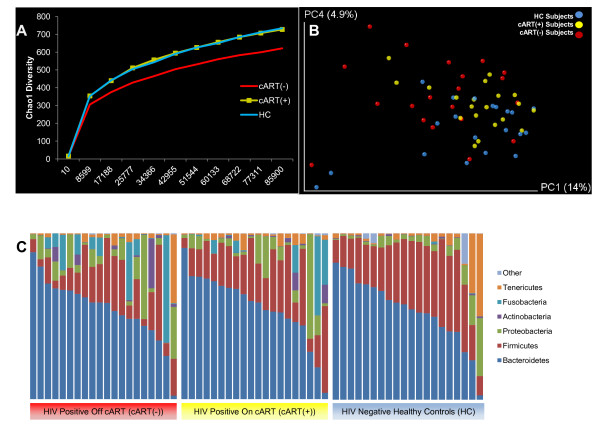
**Alpha and beta diversity. (A)** Chao1 alpha diversity indicated subjects on combination anti-retroviral therapy (cART(-)) had reduced species richness than healthy controls (HC) and subjects on (cART(+)), though only the comparison with HC was significant (*t*-test, *P* ≤0.05 at all sampling depths >17,188). Abundance curves for HC and cART(+) subjects were nearly indistinguishable. **(B)** Beta diversity was analyzed by unweighted unifrac analysis using the first and fourth principal components. These principal components were selected because principal coordinate 1 (PC1) was significantly different between cART(-) and cART(+) subjects (Kruskal-Wallis, *P* = 0.02) and both PC1 and PC4 were significantly different between cART(-) and HC subjects (Kruskal-Wallis, both *P* <0.05). HC subjects (blue) clustered relatively tightly with cART(+) subjects (yellow), whereas cART(-) subjects (red) were more diffusely scattered along PC1 and PC4 (Adonis, for cART(+) vs cART(-) *P* = 0.06, and for cART(-) versus HC *P* = 0.02). **(C)** Phylum level composition of each subject was sorted based on HIV and cART status. The abundance of Firmicutes was significantly reduced in cART(-) subjects compared with healthy subjects (analysis of variance (ANOVA), *q* = 0.06) while Fusobacteria were significantly enriched (ANOVA, *q* = 0.11).

As groups, the HC and cART(+) subjects revealed very similar alpha diversity rarefaction profiles. However, cART(-) subjects exhibited significant reduction of alpha diversity using the Chao1 diversity metric, which accurately estimates OTU richness for microbial communities [43], compared with HC, indicating HIV infection is associated with a potential collapse in alpha diversity (two tailed *t*-test, *P* ≤0.05 at all sampling depths >17,188) (Figure 2A). A similar, though insignificant trend was observed between cART(+) and cART(-) subjects (two tailed *t*-test, *P* = 0.05, 0.1 for all sampling depths >10). Similar overall trends were observed using two other alpha diversity metrics, including observed species and Phylogenetic Diversity, though neither metric yielded significant differences between any of the three cohorts, suggesting any difference in alpha diversity was minor (Additional file 3). Together, these derivative data suggest HIV infection resulted in a slight reduction of alpha diversity in cART(-) subjects that was reversed to near equivalence with HC in cART(+) subjects. Beta diversity analysis was then performed using the unweighted unifrac distance metric to determine whether HC, cART(+) or cART(-) subjects differed in their microbial composition. Adonis analysis of the resulting unifrac distance matrix suggested that the microbial compositions of: 1) cART(-) subjects were significantly different from HC subjects (*P* = 0.017); 2) cART(+) and cART(-) subjects overlapped but had slightly different trends (*P* = 0.053); and 3) cART(+) subjects were not statistically different from HC subjects (*P* = 0.1). Principal coordinate analysis (PCoA) of the first and fourth principal components of the unweighted unifrac distance matrix allowed visualization of these differences (Figure 2B). The variation captured in the first, third and fourth principal components (14%, approximately 5%, and 4% of total variation, respectively) of the unweighted unifrac analysis varied significantly between cART(-) and HC subjects (Kruskal-Wallis, all *P* ≤0.05). Excluding age (Pearson correlation with principle coordinate 1 (PC1), *P* <0.001), which is known to correlate with changes in intestinal microbiota composition [26,44,45], no other subject-reported metadata (including serum CD4 levels, serum viral titers, duration of infection, and ethnicity) significantly correlated with any of the first five principal components of the combined data. Thus, both alpha and beta diversity analysis suggested that HIV infection resulted in ecological changes relative to healthy controls that were partially normalized in cART(+) subjects.

As an initial confirmation of the beta diversity predicted differences, the microbial composition of samples from HIV-infected (both cART(+) and cART(-)) subjects was compared with that of HC subjects at the phylum level. Significant phylum level differences were corrected for multiple comparisons using a significance cutoff of *q* <0.15 [46]. Samples obtained from cART(-) subjects were enriched with Fusobacteria (ANOVA, *q* = 0.1) and depleted of Firmicutes (ANOVA, *q* = 0.058), compared to HC samples (Figure 2C). However, cART(+) subjects displayed only intermediate enrichment of Fusobacteria (ANOVA, *q* = 0.27) and depletion of Firmicutes (ANOVA, *q* = 0.27). Therefore, this analysis also suggested microbial composition in cART(-) subjects was significantly different from HC and that the composition of cART(+) was partially, though incompletely, normalized to that of HC.

### Numerous mucosal microbial taxa are differentially abundant in cART(-) subjects

**Figure 3:**
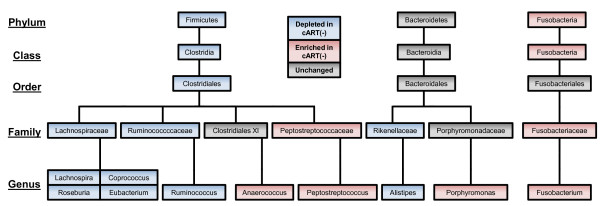
**Phylogenetic differences in subjects not on combination anti**-**retroviral therapy (cART(-)).** A phylogenetic tree populated with taxa that were significantly associated with HIV infection. Blue boxes indicate taxa that were significantly depleted in cART(-) subjects; Red boxes indicate clades or taxa that were enriched in cART(-) subjects; and grey boxes indicate clades or taxa that were neither enriched nor depleted in HIV, and are only included to contextualize taxa that were differentially abundant. All associations were determined by analysis of variance and were significant at *q* <0.15 except *Porphyromonas* (*q* = 0.155).

To identify all bacterial taxa that were differentially abundant in cART(-) subjects, OTUs were binned at every taxonomic level, thresholded such that any taxon present in fewer than 20% of samples was discarded, and analyzed for compositional differences between cART(-) and HC subjects. The identified microbial enrichments and depletions are shown according to their taxonomic assignment in Figure 3. Numerous Clostridiales genera were depleted in cART(-) subjects, including *Lachnospira*, *Coprococcus*, *Eubacterium*, *Roseburia*, *and Ruminococcus*. Furthermore, the depletion of the *Alistipes* genus from the Bacteroidetes phylum and an unclassified genus from the Veillonellaceae family was also seen. Conversely, two Clostridiales genera, *Peptostreptococcus* and *Anaerococcus*, one Bacteroidiales genus, *Porphyromonas*, and one Fusobacteriales genus, *Fusobacterium*, were enriched in cART(-) subjects compared with HCs. All enriched and depleted bacteria were significant at *q* <0.15 except the enrichment of *Porphyromonas*, which was significant at *q* = 0.157. Combined, these findings suggest that HIV infection, in the absence of cART, results in extensive microbial compositional alterations at the rectal mucosal surface.

### CART partially restores mucosal microbiota composition

**Figure 4:**
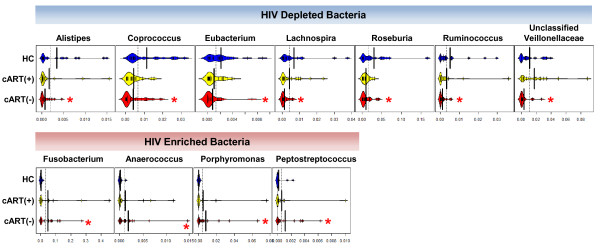
**Combination anti**-**retroviral therapy (Cart) partially normalizes microbial composition.** Genera that were significantly depleted or enriched in subjects not on cART (cART(-)) were analyzed in those on cART (cART(+)) and healthy controls (HC) to reveal the extent to which cART use reversed microbial changes. While no genera were significantly different between cART(+) and HC subjects, cART(+) subjects displayed intermediate levels of enrichment and depletion in all tested genera. The largest vertical black bars in each bean plot represent group averages. Thin vertical black bars represent at least one sample. The x-axis represents the percent abundance of genera for each sample; *q <0.15 relative to HC.

Given the systemic morbidity, mortality, and microbial changes associated with untreated HIV-1 infection, the influence of cART on microbial composition was of interest. This would ideally be tested longitudinally on subjects as they transitioned onto cART. However, given the difficulty in recruiting and retaining compliant patients, differences were compared between cART(+) and cART(-) subjects. First, the abundances of genera that were altered in cART(+) subjects were analyzed with respect to HC subjects. The genera *Roseburia* and *Coprococcus* appeared significant according to the uncorrected *P*-value (ANOVA, *P* <0.05), but no genera were significantly different in cART(+) subjects compared with HC subjects at *q* <0.15 after correction for multiple comparisons. However, many of the genera that significantly differed in cART(-) subjects were of intermediate significance in cART(+) subjects (data not shown). To allow qualitative comparison of the extent to which cART normalized the rectal microbiota in cART(+) subjects relative to cART(-) and HC subjects, bean plots of each relevant genus were generated for each subject group (Figure 4). Every genus that was enriched or depleted in cART(-) subjects was similarly, but less drastically, enriched or depleted in cART(+) subjects relative to HC subjects.

Given that long-term cART can substantially reconstitute CD4+ T cells, duration of cART might be expected to correlate with increased normalization of these bacteria. Unfortunately, durations of compliant cART could not be reliably estimated in this study. As a proxy, duration of infection of cART(+) subjects was correlated with the abundance of each genus above. However, none of the genera positively correlated significantly with duration of infection. Hence, cART appeared to partially remediate microbial composition from that in cART(-) subjects, but failed to completely restore the microbiota to levels observed in HC. This interpretation should be tempered by the proxy parameter for treatment duration and quality, and a cohort size that limits sensitivity in detecting temporal microbial changes.

### Imputed metagenomic metabolic functions significantly vary with HIV infection in the absence of cART

Having identified distinct microbial changes in cART(-) subjects compared to HC subjects, concomitant functional metagenomic differences might also be expected. Metagenomic composition is traditionally defined using shotgun sequencing. In the absence of measured metagenomic sequencing data, PICRUSt (http://picrust.github.io/picrust) in combination with HUMAnN [39] was used to bioinformatically impute sample metagenomes and determine relative genomic abundances of KEGG metabolic pathways from all subjects, respectively. PICRUSt allows imputation of most microbial genomes present in each sample based on sequence similarity of input GreenGenes sequences to sequenced reference genomes. When combined with HUMAnN, a separate bioinformatic tool that organizes metagenomic data into relative abundances of KEGG pathways per sample, the resulting data are highly comparable to sequenced metagenomic data and observed metabolomic data [26,47].

**Figure 5:**
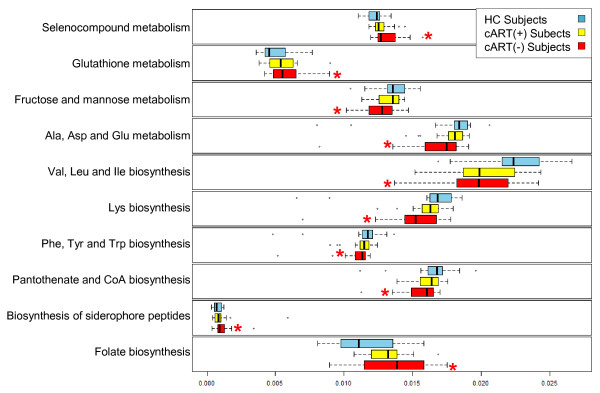
**Imputed metagenomic differences between subjects not on combination anti**-**retroviral therapy (cART(-)) and healthy control (HC) subjects.** The relative abundance of metabolic pathways encoded in each imputed sample metagenome was analyzed by HIV infection status of each subject using box plots. From these box plots, clear differences are observed between the relative abundance of several imputed metagenomic functions between cART(-) subjects and HC. Significance of each comparison was determined using Kruskal-Wallis one way analysis of variance. Box plots of subjects on cART (cART (+)) are included to provide context for each comparison. Vertical black bars represent group averages. The x-axis represents the percent abundance of pathways for each imputed sample metagenome. Whiskers represent the interquartile ranges multiplied by 1.5; *q <0.25 relative to HC.

Using these bioinformatic tools, metagenomic functions were compared between cART(-), cART(+) and HC subjects. For these metagenomic analyses, the *q*-value threshold for correcting multiple comparisons was relaxed to include *q* <0.25 comparisons in agreement with previous studies using this methodology [26]. Like the analyses above, no significant differences were observed between cART(+) and HC subjects or between cART(+) and cART(-) subjects after correction for multiple comparisons. However, 10 KEGG pathways were significantly different between cART(-) and HC subjects (Kruskal-Wallis, *q* <0.25). Plotting these pathways with respect to cART status revealed that the distribution of these pathways was similar to the distribution of enriched and depleted genera (Figure 5); While cART(-) subjects exhibited the most enrichment or depletion of each pathway, cART(+) subjects had intermediate levels of metagenomic pathway abundance relative to HC (Figure 5). Overall, the metagenomes of cART(-) subjects tended to be depleted of amino acid production, amino acid metabolism, CoA biosynthesis, and fructose/mannose metabolism compared with HC subjects. Instead, the microbiota of cART(-) subjects were metagenomically enriched for glutathione metabolism, selenocompound metabolism, folate biosynthesis and siderophore biosynthetic genes. These results indicate that HIV infection in the absence of cART results in significant functional metagenomic differences that are not fully restored with cART. Such functional differences may reflect the functions that HIV-infected mucosa select for and could have downstream implications on vitamin and nutrient availability for the host.

## Discussion

This study represents one of the first attempts to define changes in the mucosal microbiota composition in HIV infection and revealed several HIV-dependent changes in the mucosal microbiota. HIV infection in the absence of cART was associated with significant increases in *Fusobacterium*, *Anaerococcus*, *Peptostreptococcus* and *Porpyromonas* species and significant decreases in *Roseburia*, *Alistipes*, *Coprococcus*, *Eubacterium*, *Ruminococcus* and *Lachnospira* species. Surprisingly, of the genera enriched in cART(-) subjects all are (i) commonly isolated from the oral cavity and (ii) considered opportunistic pathogens. Indeed, *Anaerococcus*, *Porphyromonas* and *Peptostreptococcus* species commonly cause infections in immunocompromised individuals [48,49].

*Fusobacterium* species commonly colonize the oral cavity and are classified as pathogenic due to: 1) their ability to integrate into healthy oral streptococcal biofilms and subsequently facilitate colonization of other periodontal pathogens [50] and 2) their ability to translocate into the blood and contribute to bacteremia, preterm birth, organ abscesses and possibly coronary artery disease [51-54]. The levels of intestinal enrichment of *Fusobacterium* observed in cART(-) subjects is rare and typically only occurs in the context of severe disease, like colorectal adenoma and appendicitis [55-58]. Indeed, HIV-infected subjects are three times more likely than uninfected individuals to develop acute appendicitis, and have an increased risk of colorectal cancer despite advances in cART [59-61]. It is therefore possible that the increased mucosal concentration of *Fusobacterium* may contribute to observed increases in some co-morbidities in HIV-infected patients. Conversely, genera depleted in cART(-) subjects, including *Roseburia*, *Alistipes*, *Coprococcus*, *Eubacterium*, *Ruminococcus* and *Lachnospira* tend to be non-pathogenic. In IBD, *Roseburia* species are also commonly depleted, which is thought to be detrimental to the host due to their tendency to produce anti-inflammatory short-chain fatty acids (SCFAs) from fiber sources [26,62,63]. In the course of publication of the present study, there has been an independent report of the impact of HIV infection on intestinal mucosal microbial composition and metabolic function [64]. The findings in both studies were largely concordant, and in particular highlighted the impact of HIV infection on amino acid metabolism (most notably tryptophan catabolism), and its relevance as a modifier of mucosal immune homeostasis

This study also revealed several important, imputed metagenomic functional pathways that varied in abundance between cART(-) and HC subjects. Although previous studies have suggested that intra-subject metagenomic content tends to remain relatively stable over time independent of microbial composition, new studies suggest metagenomic content can vary in the context of certain diseases [26,41,44]. These results, combined with the observed microbial compositional changes, suggest differing metabolic functions arise based on the different microbial communities more pronounced in HIV-infected subjects. Genes encoding amino acid biosynthesis and metabolism, CoA biosynthesis, selenocompound metabolism, glutathione metabolism and folate biosynthesis were compositionally altered in cART(-) subjects. This imbalance might indicate that free vitamins and nutrients available to HIV-infected hosts might be altered. Interestingly, siderophore biosynthetic genes were enriched in cART(-) subjects. Siderophores act as quorum sensing molecules for gram-negative organisms, so the enrichment of this pathway could be indicative of increased intra- or inter-species communication in cART(-) subjects. cART(-) subjects encoded fewer genes for fructose and mannose metabolism, which may be reflective of altered environmental nutrient availability or the differing metabolic potential of bacterial species that best adapt to such environments. Besides illuminating the metabolic potential of the underlying bacterial community, these pathways might be exploitable to help normalize microbial composition of HIV-infected subjects if microbial remediation strategies prove warranted. Given the role of diet in driving microbial composition, one could imagine exploiting such differences in metabolic function by dietary optimization to enrich for preferred bacteria [65-67].

## Conclusions

Significant functional and compositional differences in rectal microbiota were observed between cART(-) and HC subjects; these were incompletely normalized by cART in cART(+) subjects. However, an HIV-associated reduction in alpha diversity was adequately normalized by cART. Phylogenetic profiles of cART(-) subjects reflected enrichment for opportunistic pathogens and depletion of nonpathogenic genera. The differing imputed metagenomic compositions between cART(-) subjects and HC subjects suggested HIV infection altered the rectal ecosystem and selected for different microbial metagenomic functions.

## Abbreviations

ANOVA: Analysis of variance; cART: Combination anti-retroviral therapy; cART(+): HIV-positive subjects receiving combination anti-retroviral therapy; cART(-): HIV-positive subjects not receiving combination anti-retroviral therapy; HC: Healthy control; HUMAnN: HMP unified metabolic analysis network; IBD: Inflammatory bowel disease; IL: Interleukin; KEGG: Kyoto encyclopedia of genes and genomes; NRTI: Nucleotide and nucleoside reverse transcriptase inhibitor; NNRTI: Non-nucleoside reverse transcriptase inhibitor; OTU: Operational taxonomic unit; PBMC: Peripheral blood mononuclear cell; PC: Principal component; PCoA: Principal coordinate analysis; PCR: Polymerase chain reaction; PI: Protease inhibitor; PICRUSt: Phylotypic investigation of communities by reconstruction of unobserved states; QIIME: Quantitative Insights into Microbial Ecology; SCFA: Short chain fatty acid.

## Competing interests

The authors declare that they have no competing interests.

## Authors’ contributions

JBr, PA and IM designed the study format. PA coordinated the sample collection. IM and XL pre-processed samples and coordinated sample storage. IM, JBr, and MT designed the analytic strategies. PR and JBo generated sequencing data. IM, MT, JBo, PR, JJ and JBr analyzed data. IM performed computational analysis. All authors interpreted data. IM, JBr and PA drafted the manuscript. All authors read and approved the final manuscript.

## Supplementary Material

Additional file 1 **Primers used for the V4 rRNA PCR and sequencing analysis.** Contains three tables containing: (1) Reverse PCR primers used in the Illumina-based high-throughput sequence analysis of bacterial 16S rRNA genes; (2) Forward PCR primer used in the Illumina-based high-throughput sequence analysis of bacterial 16S rRNA genes; and (3) Sequencing primers used in the Illumina-based high-throughput sequence analysis of bacterial 16S rRNA genes. PCR, polymerase chain reaction. Click here for file

Additional file 2 **Individual anti-retroviral drugs taken by subjects on combination anti-retroviral therapy (cART(+)).** Each cART(+) received an average of 3.65 specific drugs that are tabulated here. Click here for file

Additional file 3 **Additional metrics of alpha diversity.** Observed species (A) and Phylogenetic Diversity (B) are independent metrics of alpha diversity. Both indicated that subjects not on combination anti-retroviral therapy (cART(-)) exhibited reduced alpha diversity relative to both healthy controls (HC) and those on cART (cART(+)), though these differences were not statistically significant using either metric. In both cases, the alpha diversity curves of HC subjects and cART(+) subjects were nearly indistinguishable. Click here for file
