# Supplementary figures and images for: HIV Infection is associated with compositional and functional shifts in the rectal mucosal microbiota
Source: Microbiome. 2013 Oct 12;1:26. doi: 10.1186/2049-2618-1-26 (PMC3971626; doi:10.1186/2049-2618-1-26)

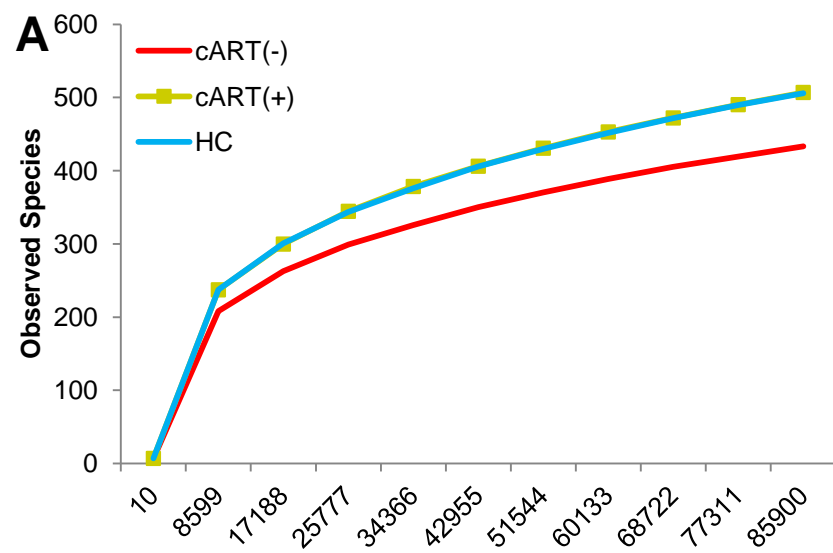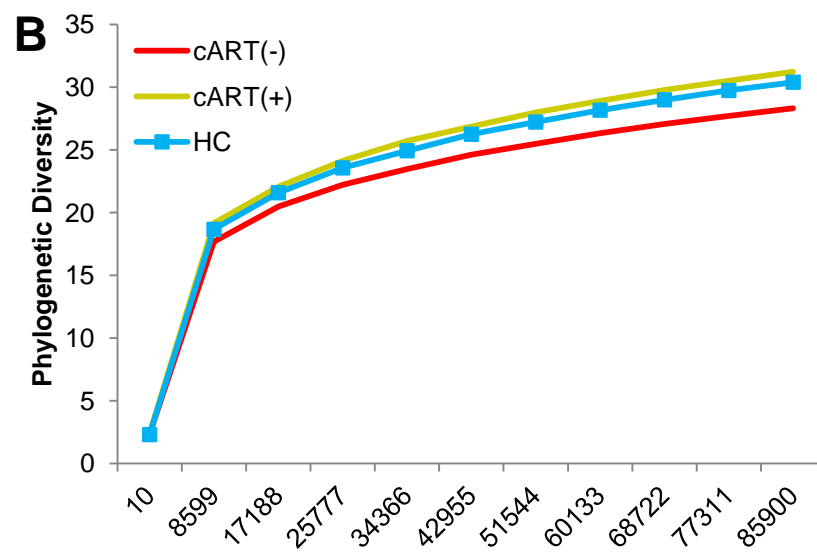

Supplement: Additional file 3 — Additional metrics of alpha diversity. Observed species (A) and Phylogenetic Diversity (B) are independent metrics of alpha diversity. Both indicated that subjects not on combination anti-retroviral therapy (cART(-)) exhibited reduced alpha diversity relative to both healthy controls (HC) and those on cART (cART(+)), though these differences were not statistically significant using either metric. In both cases, the alpha diversity curves of HC subjects and cART(+) subjects were nearly indistinguishable. [file 2049-2618-1-26-S3.pdf]
